# Effects of different categories of tea powders incorporated into fresh noodles on quality, non-volatile metabolites, and aroma characteristics

**DOI:** 10.1016/j.fochx.2026.104202

**Published:** 2026-07-13

**Authors:** Haozhen Li, Xiangjun Chen, Shuyao Wang, HuiHui Zhao, Yi Xie, Long Yang

**Affiliations:** a College of Plant Protection, Agricultural Big-Data Research Center and Key Laboratory of Agricultural Film Application of Ministry of Agriculture and Rural Affairs, Shandong Agricultural University, Tai'an 271018, China; b Bioresource Engineering, McGill University, Sainte-Anne-de-Bellevue, QC H9X 3V9, Canada; c Ri Zhao Cha Cang Tea Co. Ltd, Rizhao 276800, China

**Keywords:** Fresh noodles, Tea powder, Non-volatile metabolites, Aroma characteristics, Flavonoid biosynthesis

## Abstract

Tea powder incorporation is a promising strategy for developing functional fresh noodles, but the effects of different tea powders on noodle quality and metabolic characteristics remain unclear. In this study, 20% of wheat flour was replaced with four tea powders, namely matcha, chenpi-polygonatum tea, rose black tea, and jasmine tea, to evaluate changes in structural characteristics, quality traits, and both volatile and non-volatile metabolites. Tea powder promoted a denser starch-gluten matrix, thereby improving cooking performance, texture, and overall sensory quality and acceptability, while significantly increasing starch and phenolic contents, as well as *in vitro* antioxidant activity. Non-volatile metabolomics showed that differential metabolites were mainly enriched in flavonoid biosynthesis and flavone and flavonol biosynthesis. Volatile profiles shifted from a simple cereal-like aroma to more complex floral, fruity, and fresh notes, with jasmine tea showing the strongest aroma-enhancing effect. These results provide support for the development of functional tea powder-enriched fresh noodles.

## Highlights

• Tea powders improved noodle texture, cooking quality, and matrix compactness.
• Flavonoid pathways dominated non-volatile metabolic variation.
• Tea powders redirected aroma toward floral, fruity and fresh notes.
• Jasmine tea powder produced the strongest aroma-enhancing effect.

## Graphical abstract

**Figure:**
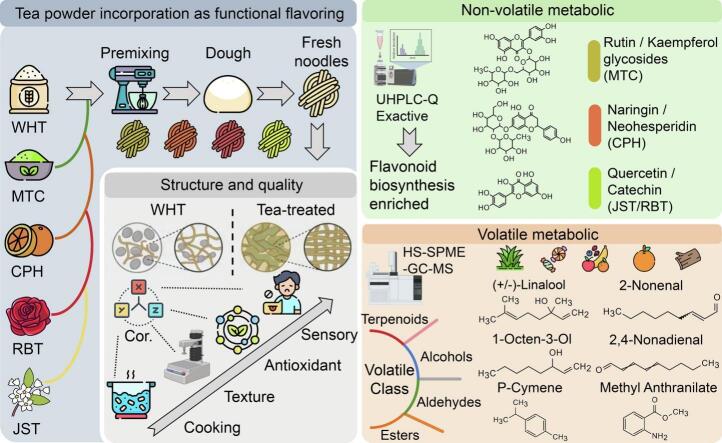

## 1 Introduction

Fresh noodles are a widely consumed wheat-based staple food, and their cooking stability, textural properties, color, and aroma jointly determine their eating quality and commercial value. In recent years, staple food processing has increasingly moved toward natural, nutritional, and functional development. Consequently, plant-based ingredients rich in bioactive compounds and possessing distinctive flavor characteristics have attracted growing attention (Wang & Jian, 2022). Tea powders are widely available, possess distinctive color and aroma characteristics, and contain tea polyphenols, flavonoids, and various aroma precursors. Their application in noodle products not only helps increase the content of functional components but also affects the sensory characteristics of the final products. The incorporation of tea-derived ingredients into fresh noodle formulations has been associated with improved product quality (Wang et al., 2023). Meanwhile, different tea powders vary considerably in raw material origin and processing methods, and these differences are further reflected in their sensory attributes and chemical composition. For example, the flavonoid composition and astringency of matcha fluctuate with cultivar and processing conditions, whereas jasmine tea exhibits more pronounced aroma expression after multiple rounds of scenting (Li, Song, Zhang, Hou, & Yang, 2025; Xue et al., 2025). Therefore, differences among tea powder types may further influence their quality performance and flavor characteristics in fresh noodles.

After being incorporated into fresh noodles, tea powders undergo water absorption, mixing, sheeting, and cooking together with flour components, and their effects are ultimately reflected in the internal structure and eating quality of the noodles. During dough mixing, the redistribution of water influences gluten network development, whereas changes in hardness during cooking are strongly related to moisture distribution, starch properties, and overall structural changes. Enhanced protein-starch interactions caused by thermal treatment are often accompanied by increases in hardness, elasticity, and chewiness (An et al., 2024; Dufour et al., 2023; Liu et al., 2024). In tea powder-enriched fresh noodles, tea polyphenols, organic acids, pigments, and aroma precursors enter the starch-gluten matrix and jointly participate in the formation of texture, cooking tolerance, and sensory quality. Accordingly, the differences caused by different tea powders are not limited to cooking loss or hardness changes but may also involve internal structural changes and the migration of chemical components.

Differences in the composition of non-volatile metabolites in tea powders may further affect their quality performance and functional characteristics in fresh noodles. During tea processing, flavonoids and their glycosylated derivatives, catechins, amino acids, phenolic acids, and organic acids undergo continuous changes and jointly contribute to the formation of sensory attributes such as bitterness, freshness, and mellowness. Quantitative non-volatile sensometabolomics studies have shown that the chemical differences among tea samples with different taste profiles are mainly concentrated in flavonoids, amino acids, and related compounds (Shan et al., 2025). Processing studies have further demonstrated that non-volatile constituents such as flavonoid glycosides change dynamically as processing proceeds and are closely associated with final quality formation (Li, Song, Zhang, Wang, & Yang, 2025). Metabolomics combined with sensory analysis has also confirmed a close relationship between changes in non-volatile components and taste quality (Ni et al., 2025). Therefore, after different tea powders are applied to fresh noodles, differences may exist in their non-volatile metabolite composition and metabolic pathways.

In addition to non-volatile components, volatile metabolites are also important factors affecting the flavor characteristics of tea powder-enriched fresh noodles. Under different raw material characteristics, cultivar backgrounds, and processing conditions, the dominant categories of volatile compounds and aroma expression in tea vary markedly, leading to differentiated flavor characteristics (Wang et al., 2025; Zhang, Gu, et al., 2025). Volatile metabolomics, together with odor activity value (OAV) assessment, has been widely applied in tea aroma research to pinpoint key aroma-active compounds (Wang, Liang, et al., 2024). In scented tea processing, the scenting process can promote the accumulation of volatiles and aroma remodeling, while different flower types or scenting conditions may further influence aroma intensity and aroma persistence (Pang et al., 2025). Compounds including linalool, methyl salicylate, geraniol, methyl benzoate, benzyl alcohol, and indole play important roles in shaping tea aroma (Hua et al., 2024; Jiang et al., 2025). Therefore, tea powders differing in origin and processing pathways may vary in the accumulation levels of volatiles, dominant categories, and key aroma-active compounds, thereby further influencing the flavor performance of fresh noodles.

Based on the above, this study used wheat fresh noodles as the research object and selected matcha, chenpi-polygonatum tea, rose black tea, and jasmine tea powders to prepare fresh noodle samples by replacing 20% of wheat flour with each tea powder. The main research contents included: (a) comparing the effects of different tea powders on the microstructure, cooking properties, texture, and sensory quality of fresh noodles; (b) determining the contents of starch, total phenols, and total flavonoids as well as *in vitro* antioxidant activity, to analyze changes in the physicochemical properties and antioxidant capacity of fresh noodles; (c) characterizing differences in non-volatile metabolites and their enriched pathways using LC–MS; and (d) analyzing differences in volatile metabolite composition and screening key differential volatile compounds by combining HS-SPME-GC–MS with aroma activity evaluation. This study is expected to provide a reference for raw material selection, quality improvement, and flavor regulation of functional tea powder-enriched fresh noodles.

## 2 Materials and methods

### 2.1 Materials and reagents

Commercial wheat flour purchased from Yihai Kerry Food Marketing Co., Ltd. (China) was used as the raw material for noodle preparation. The wheat flour contained 73.0 g/100 g starch, 11.0 g/100 g protein, and 1.6 g/100 g fat. Matcha, chenpi–polygonatum tea, rose black tea, and jasmine tea were all commercial Grade I food-grade tea products provided by Rizhao Chacang Tea Co., Ltd. (Rizhao, China). According to the supplier information, all tea materials were ground and passed through a 100-mesh sieve before noodle preparation to ensure uniform dispersion in the wheat flour matrix. The corresponding reference standards were selected according to the product category of each tea powder: matcha powder was referenced to GB/T 34778–2017, chenpi–polygonatum tea powder was referenced to GH/T 1091–2014, rose black tea powder was referenced to GH/T 1247–2019, and jasmine tea powder was referenced to GB/T 22292–2017. More detailed information on the appearance/color description, representative chemical characteristics, and sensory attributes of the four tea powders is provided in Table S1. LC–MS-grade methanol, acetonitrile, formic acid, and acetic acid were purchased from Merck (Darmstadt, Germany). Ammonium formate, ammonium acetate, and the n-alkane standard mixture (C7–C40) were obtained from Sigma-Aldrich (St. Louis, MO, USA). Sodium chloride was supplied by Sinopharm Chemical Reagent Co., Ltd. (Shanghai, China). Ultrapure water was prepared using a Milli-Q system. Reference standards were used for targeted quantitative validation of selected non-volatile metabolites. All other reagents were of analytical grade.

### 2.2 Preparation of fresh noodles

**Fig. 1:**
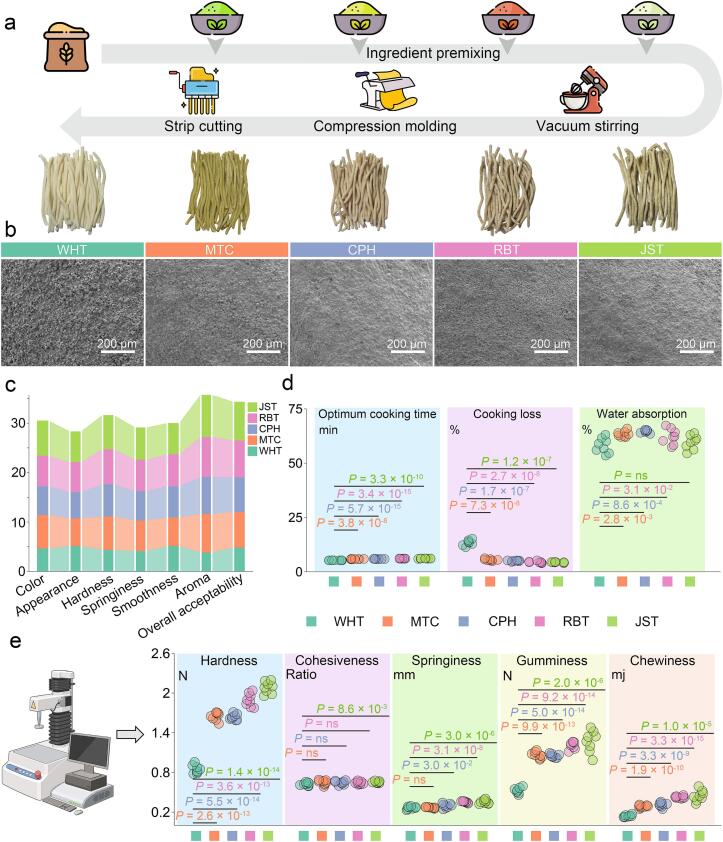
Preparation process, microstructure, sensory quality, cooking properties, and textural characteristics of noodles with different tea powder treatments. (a) Schematic diagram of noodle preparation. (b) SEM images of noodle cross-sections. (c) Sensory evaluation. (d) Cooking properties. (e) Texture profile analysis (TPA).

Fresh noodles were prepared using a vacuum dough mixer (Yangzi, China). Five treatments were designed: WHT (control, without tea powder), MTC (with matcha powder), CPH (with chenpi–polygonatum tea powder), RBT (with rose black tea powder), and JST (with jasmine tea powder). For the tea-treated groups, tea powder was used to replace 20% (*w*/w) of wheat flour. Accordingly, the control formulation consisted of 100 g wheat flour, 30 g purified water, and 0.6 g salt, whereas the tea-treated formulations consisted of 80 g wheat flour, 20 g tea powder, 30 g purified water, and 0.6 g salt. The 20% replacement level was selected as a representative level for comparative evaluation among different tea powders based on previous studies on tea-type powder and plant functional powder-enriched noodles (Li, Xu, Chen, Ding, et al., 2025; Han et al., 2022). Wheat flour was first mixed with the corresponding tea powder and then transferred into the vacuum dough mixer. Purified water containing dissolved salt was added, and the mixture was kneaded to obtain a homogeneous dough. The dough was subsequently sheeted and cut into fresh noodles (Fig. 1a). The prepared noodles were cylindrical in shape, with a diameter of approximately 2.0 mm, and were used for subsequent analyses.

### 2.3 Microstructure analysis

Freeze-dried noodle samples were placed on aluminum stubs with conductive tape, coated with gold, and observed under a scanning electron microscope (SEM; Regulus, Hitachi, Japan) at 5.0 kV and a working distance of 8.0 mm. The images obtained were used to characterize the noodle microstructure.

### 2.4 Sensory evaluation

The sensory quality of cooked noodle samples was evaluated with reference to GB/T 25005–2010, Sensory analysis-Methods for sensory evaluation of instant noodles, and GB/T 10220–2012, Sensory analysis-Methodology-General guidance, with slight modifications according to the characteristics of cooked fresh noodles and recent studies on noodle sensory evaluation (Cao et al., 2024; Tang et al., 2024). The samples were cooked according to their optimum cooking time prior to sensory evaluation. The sensory panel consisted of 12 assessors, including 6 males and 6 females. Before formal evaluation, all assessors were informed of the evaluation procedure, attribute definitions, and scoring criteria. Color, appearance, hardness, springiness, smoothness, aroma, and overall acceptability were selected as the evaluation attributes. A 9-point hedonic scale was used for sensory scoring, ranging from 1 (dislike extremely) to 9 (like extremely). Detailed scoring criteria for each sensory attribute are provided in Supplementary Table S2. All samples were presented to the panelists in a randomized order using three-digit codes, and the final sensory scores were expressed as the mean values of the 12 assessors. Detailed information regarding the ethical statement is provided in the Ethical Statements section.

### 2.5 Cooking properties

During cooking in boiling deionized water, one noodle strand was removed every 10 s and lightly pressed between two transparent glass plates. The disappearance of the white core in the strand center was taken as the optimum cooking time.

For measurement of water absorption, 20.0 g of noodle sample was boiled in 400 mL of deionized water for the optimum cooking time. The cooked noodles were then taken out, allowed to cool to room temperature, and gently dabbed with filter paper to remove surface moisture prior to weighing. The initial and final weights were denoted as W1 and W2, respectively, and the water absorption rate was calculated using Eq. (1):

$$\mathrm{WAR}\;\left(\%\right)=\left(\frac{W_{2}-W_{1}}{W_{1}}\right)\times 100$$

For cooking loss determination, the cooking liquor was collected and transferred into a pre-weighed evaporating dish, and then dried at 105 °C to constant weight. The mass of the residue was recorded as W3. Cooking loss was calculated using Eq. (2):

$$\mathrm{CL}\;\left(\%\right)=\left(\frac{W_{3}}{W_{1}}\right)\times 100$$

where W1 is the sample weight before cooking, W2 is the sample weight after cooking, and W3 is the mass of the dried residue in the cooking water. All measurements were performed in eight replicates.

### 2.6 Texture profile analysis

Textural properties of cooked noodles were measured using a TA-XT Plus texture analyzer (Stable Micro Systems, UK). After cooking to the optimum time, the noodles were removed and gently blotted to eliminate surface moisture. Three strands were placed side by side on the platform and analyzed in TPA mode with a two-cycle compression test. The operating parameters were set at a pre-test speed of 1 mm/s, a test speed of 2 mm/s, a post-test speed of 1 mm/s, a compression ratio of 80%, and a trigger force of 5.0 g. Each sample was tested eight times.

### 2.7 Starch composition, phenolic contents, and antioxidant activity

The contents of amylose, amylopectin, and total starch in the samples were determined based on the specific binding property of concanavalin A toward amylopectin but not amylose, thereby enabling the separation of amylose and amylopectin. The starch was then hydrolyzed into glucose by a starch-hydrolyzing enzyme system, and the contents were calculated by measuring the glucose concentration. Total phenolics were quantified by the Folin–Ciocalteu assay based on the formation of a blue chromogenic product under alkaline conditions, with absorbance recorded at 760 nm. Total flavonoids were measured using the NaNO_2_-Al(NO_3_)_3_-NaOH colorimetric assay, in which flavonoids formed a colored complex with aluminum ions and were detected at 510 nm. Antioxidant activity was assessed by DPPH and ABTS radical scavenging assays, with absorbance monitored at 517 and 734 nm, respectively. Ferric reducing antioxidant power was evaluated by the Fe^3+^-TPTZ reduction assay and expressed according to the absorbance at 590 nm. All measurements were carried out in quadruplicate. All measurements were performed according to the protocols provided with the Byabscience kits.

### 2.8 LC-MS detection and data analysis

Approximately 100 mg of sample was extracted in a centrifuge tube with 800 μL of precooled methanol/water (4:1, *v*/v) containing internal standards and grinding beads. After low-temperature grinding and ultrasonic extraction, the samples were kept at −20 °C and centrifuged at 4 °C, and the supernatants were collected for analysis. A UHPLC-Q Exactive high-resolution mass spectrometer was used for metabolite analysis in both positive and negative ion modes, with data acquired across *m*/*z* 70–1050. Raw data were processed by Progenesis QI for peak extraction, alignment, and integration to generate a metabolite feature matrix, and metabolite annotation was performed using the HMDB and METLIN databases. After data preprocessing, including missing value handling, normalization, and log transformation, PCA and OPLS-DA were used for multivariate analysis. Differential metabolites were identified based on VIP > 1 and *p* < 0.05, and then subjected to KEGG annotation and enrichment analysis. Each treatment group included six biological replicates in the LC-MS analysis. Targeted quantification of selected phenolic/flavonoid-related metabolites was performed using an external standard method with reference compounds according to (Li et al., 2025b). Each treatment group was analyzed with three biological replicates.

### 2.9 HS-SPME-GC–MS analysis and data processing

A 3 g noodle sample was placed in a 20 mL headspace vial, mixed with internal standard and saturated sodium chloride solution, and sealed immediately. Volatile metabolites were determined by HS-SPME-GC–MS. After headspace extraction, the absorbed analytes were introduced into the instrument, separated on a capillary column, and analyzed by mass spectrometry in full-scan mode using an EI source. The raw data were extracted and processed by software to generate a feature matrix reflecting the relative abundance of volatile metabolites in sample, and compounds were annotated based on retention index and database information. PCA and OPLS-DA were used to characterize multivariate differences among samples, and metabolites meeting the thresholds of VIP > 1 and *p* < 0.05 in Student's *t*-test were regarded as differential metabolites. KEGG database-based pathway annotation and enrichment analysis were further conducted to identify the related metabolic pathways involving the differential volatile metabolites. Each treatment group included six biological replicates in the HS-SPME-GC–MS analysis.

### 2.10 Statistical analysis

Results are expressed as mean ± SD. Statistical differences among groups were evaluated using one-way ANOVA, followed by multiple comparisons. Differences were considered significant at *p* < 0.05. Bar plots were prepared with GraphPad Prism 8.0.2. Metabolomics data were analyzed by OPLS-DA using SIMCA 14.1. Heatmaps were generated using the Chiplot online platform, and network visualization was performed using Cytoscape 3.10.2.

## 3 Results and discussion

### 3.1 Microstructure and quality characteristics of noodles

As shown in Fig. 1b, the SEM images of noodle cross-sections clearly revealed the effect of tea powder incorporation on noodle microstructure. The WHT sample exhibited a relatively loose gluten–starch matrix, with exposed starch granules and numerous micropores, indicating limited encapsulation of starch by the gluten network. In contrast, all tea powder-treated samples showed a more compact matrix structure to varying degrees. In particular, RBT and MTC presented a more continuous gluten network, with starch granules more deeply embedded in the protein matrix, whereas CPH and JST showed almost no free starch granules and a tighter starch–protein interface. Overall, tea powder incorporation reduced pore density and improved matrix compactness. Similar microstructural changes have been reported in noodle enriched with tea-derived components (Chen et al., 2021; Han et al., 2020). These changes may be related to the interactions of tea polyphenols and polysaccharides with gluten proteins, as well as their effects on water distribution during dough formation, thereby promoting the development of a more integrated matrix (Dufour et al., 2023; Guo et al., 2026; Li et al., 2017; Zhang et al., 2023).

The sensory results were generally consistent with the microstructural observations. Compared with WHT, all tea powder-treated noodles received higher scores for color, appearance, hardness, springiness, smoothness, aroma and overall acceptability (Table S3) (Fig. 1c). Specifically, color, appearance and smoothness increased by 27.29%–54.59%, 4.92%–21.46%, and 14.76%–27.95%, respectively. Hardness and springiness increased by 50.12%–63.51% and 45.10%–59.31%, indicating that tea powder addition improved both visual quality and eating texture. More notably, aroma scores increased from 3.83 to 7.50–8.50, while overall acceptability increased by 44.93%–62.11%. Among the samples, JST achieved the highest scores in color, appearance, aroma, and overall acceptability, whereas RBT performed better in hardness and smoothness. These results indicate that tea powder not only imparted desirable color and tea-like aroma, but also improved the textural perception of noodles. Such improvements are likely associated with the formation of a more continuous starch–gluten matrix and the contribution of tea-derived volatile compounds to aroma quality (Luo et al., 2025; Wang et al., 2023; Yang et al., 2025). Although tea powder changed the original color of wheat noodles, the improved sensory color scores indicated that this color change was acceptable under the present formulation.

The cooking properties further confirmed the positive effect of tea powder on noodle quality. Compared with WHT, the optimum cooking time significantly increased from 5.18 min to 5.74–5.98 min (*P* < 0.05), with the greatest increases observed for JST (15.44%) and RBT (15.06%) (Fig. 1d). Meanwhile, cooking loss significantly decreased from 13.30% to 4.25%–5.38% (*P* < 0.05), again with JST and RBT showing the most pronounced reductions. In terms of water absorption, MTC, CPH and RBT were significantly higher than WHT (*P* < 0.05), increasing from 57.97% to 62.17%–64.53%. These results suggest that tea powder enhanced cooking tolerance and structural stability, while effectively limiting the leaching of soluble solids during cooking. This may be attributed to the strengthened matrix formed in the presence of tea-derived components, which restricted excessive starch swelling and component loss during heating (Hu et al., 2024; Kraithong et al., 2023; Liu et al., 2024; Zhang et al., 2024).

Texture profile analysis showed a similar trend. Compared with WHT, noodle hardness significantly increased from 0.84 to 1.63–2.08 (*P* < 0.05), with JST and RBT showing the largest increases (Fig. 1e). Cohesiveness changed only slightly, and a significant increase was observed only for JST. Springiness in CPH, RBT and JST was significantly higher than that in WHT (P < 0.05), increasing from 0.26 to 0.29–0.35. In addition, gumminess and chewiness both increased significantly (P < 0.05), with gumminess rising from 0.52 to 1.05–1.27 and chewiness increasing from 0.14 to 0.28–0.45. JST showed the best overall textural performance, followed by RBT. These results agree well with the SEM observations, indicating that the more compact and continuous matrix contributed to improved resistance to deformation and better chewing properties. Similar relationships between water distribution, matrix compactness and noodle texture have also been reported previously (Li et al., 2024). The improvement in sensory quality was closely related to the enhanced cooking stability and textural properties of tea powder-treated noodles. The more compact starch–gluten matrix reduced cooking loss and improved hardness, springiness, gumminess, and chewiness, thereby contributing to better eating texture and higher sensory scores. These results also indicate that tea powder did not reduce noodle quality stability under the tested conditions, but showed good compatibility with the wheat flour matrix at the selected replacement level.

### 3.2 Physicochemical properties and antioxidant capacity of noodles

**Fig. 2:**
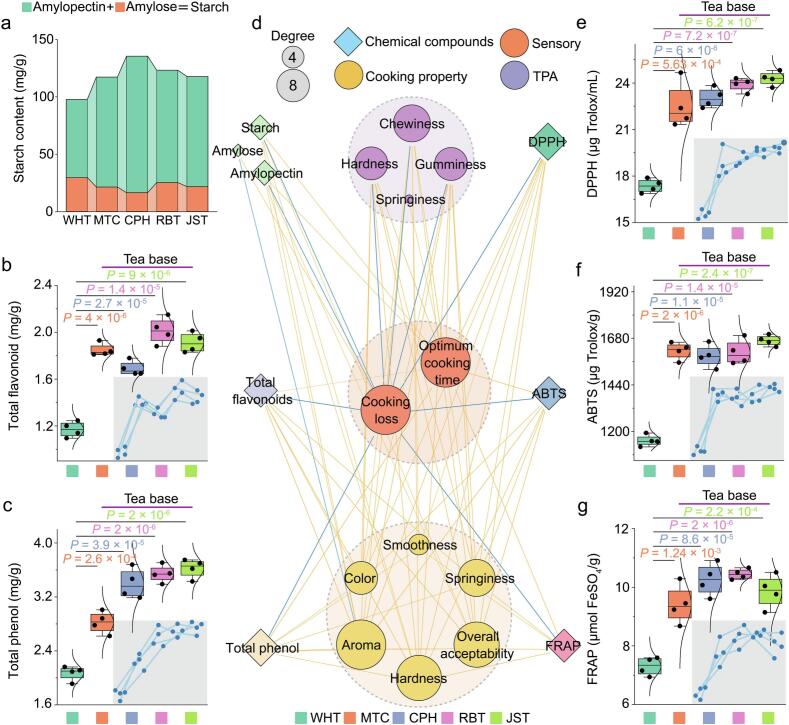
Effects of tea powder incorporation on starch composition, phenolic compounds, antioxidant capacity, and their correlations with noodle quality attributes. (a) Amylose and amylopectin contents. (b) Total flavonoid content. (c) Total phenolic content. (d) Correlation analysis among starch composition, phenolic compounds, antioxidant capacity, cooking properties, textural parameters, and sensory attributes. (e) DPPH radical scavenging activity. (f) ABTS radical scavenging activity. (g) Ferric reducing antioxidant power (FRAP).

In addition to sensory and processing quality, tea powder also affected the physicochemical composition and functional properties of noodles. Compared with WHT (97.63 ± 1.33 mg/g), all tea powder-treated samples showed higher measured total starch contents (Fig. 2a), with CPH presenting the highest value (135.33 ± 4.13 mg/g), followed by RBT (123.12 ± 3.04 mg/g), JST (117.63 ± 2.92 mg/g) and MTC (117.13 ± 2.03 mg/g). Changes were also observed in starch fractions. The amylose content decreased from 29.61 ± 0.87 mg/g in WHT to a range of 16.42 ± 0.91 mg/g to 25.27 ± 1.17 mg/g in the tea powder-treated groups, whereas amylopectin increased from 68.01 ± 1.96 mg/g to a range of 95.76 ± 2.31 mg/g to 118.91 ± 4.26 mg/g, with the highest value again observed in CPH. These results suggest that tea powder addition was accompanied by a shift in starch composition, which may influence starch swelling and gelatinization behavior during cooking and thereby affect noodle cooking stability and texture (Yao et al., 2023; Zhao et al., 2025).

Tea powder addition also significantly increased the phenolic content and antioxidant capacity of noodles. Compared with WHT (total flavonoids, 1.17 ± 0.07 mg/g; total phenolics, 2.07 ± 0.11 mg/g), all tea powder-treated samples showed higher levels of phenolic compounds, with total flavonoids and total phenolics increasing by 45.0–72.0% and 36.0–75.0%, respectively, relative to WHT (Fig. 2b, c). This enrichment was accompanied by enhanced antioxidant activity. The DPPH, ABTS and FRAP values of the tea powder-treated groups were all higher than those of WHT, increasing from 17.37 ± 0.44 μg Trolox/mL, 1152.85 ± 29.81 μg Trolox/g and 7.30 ± 0.31 μmol FeSO_4_/g to a range of 22.53 ± 1.49 μg Trolox/mL to 24.28 ± 0.46 μg Trolox/mL, 1588.86 ± 58.71 μg Trolox/g to 1668.33 ± 27.13 μg Trolox/g, and 9.41 ± 0.67 μmol FeSO_4_/g to 10.44 ± 0.18 μmol FeSO_4_/g, respectively (Fig. 2e-g). These results demonstrate that tea powder improved the functional properties of noodles. Similar improvements in the bioactive profile and quality-related attributes of noodle systems after tea material incorporation have also been reported previously (Guo et al., 2026; Han et al., 2020).

Correlation analysis further supported the relationship between phenolic enrichment and cooking performance (Fig. 2d). Total flavonoids and total phenolics were significantly positively correlated with optimum cooking time (*r* = 0.89 to 0.93) and negatively correlated with cooking loss (*r* = −0.89 to −0.90). Similarly, DPPH, ABTS and FRAP showed positive correlations with optimum cooking time (*r* = 0.84 to 0.94) and negative correlations with cooking loss (*r* = −0.88 to −0.95). These results suggest that samples with higher phenolic content and antioxidant capacity tended to exhibit better cooking stability, as reflected by longer optimum cooking time and lower cooking loss. A plausible explanation is that tea-derived phenolics interacted with gluten proteins and contributed to a more stable matrix, thereby reducing the migration of soluble components during cooking, which is consistent with previous reports (Liu et al., 2023; Tan et al., 2025).

Starch composition was also associated with cooking performance. Amylose was positively correlated with cooking loss (*r* = 0.69), whereas amylopectin was negatively correlated with cooking loss (*r* = −0.80) and positively correlated with optimum cooking time (*r* = 0.72). The trend of total starch was generally consistent with that of amylopectin. These findings indicate that starch fraction distribution may contribute to differences in cooking stability among samples. In general, a higher amylose proportion was associated with greater solid loss during cooking, whereas a higher amylopectin proportion was associated with lower cooking loss and improved cooking tolerance, which agrees with previous reports on the role of starch composition in noodle cooking behavior (Ren et al., 2025).

The differences in cooking quality were further reflected in textural and sensory attributes. Optimum cooking time was positively correlated with hardness, springiness, gumminess and chewiness (*r* = 0.67 to 0.94), whereas cooking loss was negatively correlated with hardness, gumminess and chewiness (*r* = −0.81 to −0.92), indicating that samples with better cooking stability also tended to have stronger structural support and better chewing properties. In addition, sensory attributes including color, hardness, springiness, smoothness, aroma and overall acceptability were positively correlated with hardness, gumminess and chewiness (r = 0.69 to 0.95), suggesting a general consistency between instrumental texture and sensory perception. These sensory indices were also positively correlated with total flavonoids, total phenolics, total starch, amylopectin, DPPH, ABTS and FRAP (*r* = 0.61 to 0.94). Taken together, improved cooking stability was generally accompanied by better textural performance and higher sensory acceptability in tea powder-treated noodles. The correlation analysis suggested that tea-derived non-volatile components also participated in noodle quality formation. Phenolic/flavonoid-related components introduced by tea powder may interact with gluten proteins and starch, improve matrix stability, and thereby contribute to better cooking quality, texture, and sensory acceptability.

### 3.3 Non-volatile metabolic characteristics of noodles

**Fig. 3:**
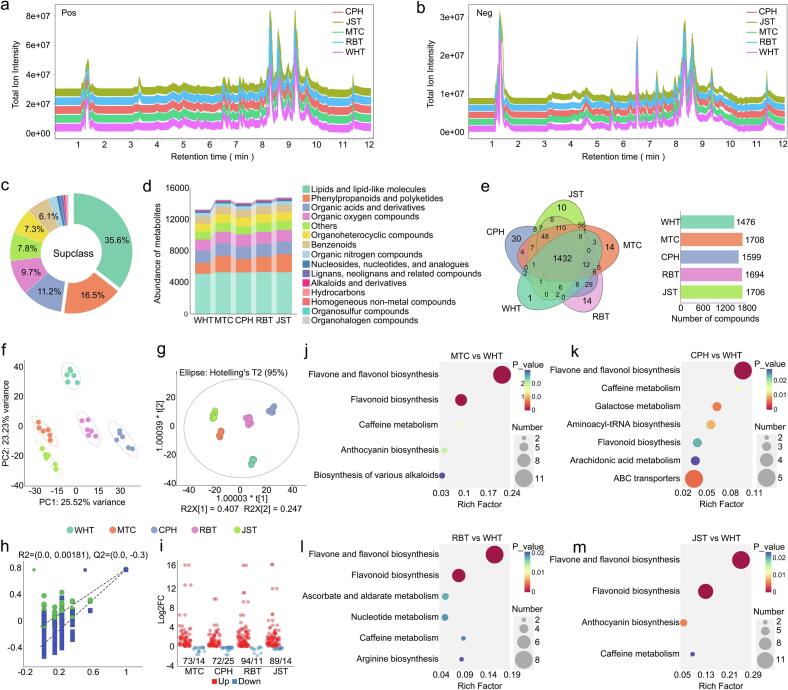
Non-volatile metabolite characteristics of noodles with different tea powder treatments. (a) Metabolic profile in positive ion mode. (b) Metabolic profile in negative ion mode. (c) Classification of identified metabolites. (d) Relative abundance changes of major metabolite classes. (e) Venn diagram of metabolites detected in different groups. (f) PCA score plot. (g) OPLS-DA score plot. (h) Permutation test of the OPLS-DA model. (i) Differential metabolites screened with VIP > 1. (j–m) KEGG pathway enrichment analysis of differential metabolites in pairwise comparisons between WHT and tea powder-treated groups.

To further reveal the intrinsic mechanism by which tea powder affects noodle quality formation, it is necessary to analyze the metabolic composition characteristics at the level of non-volatile metabolites. A total of 746 metabolites were identified in the positive ion mode (Fig. 3a) and 807 metabolites in the negative ion mode (Fig. 3b) by UHPLC-Q Exactive. The spectral characteristics differed obviously between the two ion modes, and compared with WHT, all tea powder-treated groups showed richer signal responses.

After classification of the identified metabolites, the metabolites detected in noodles were assigned to 15 categories. Among them, lipids and lipid-like molecules (35.6%), phenylpropanoids and polyketides (16.5%), and organic acids and derivatives (11.2%) were the major categories, together accounting for more than 60% of the total (Fig. 3c). Compared with WHT, tea powder treatments generally increased the relative abundance of metabolites, among which phenylpropanoids and polyketides showed the most obvious increase, rising significantly by 24.18%–67.76% relative to WHT (Fig. 3d). The Venn diagram further showed that 1432 metabolites were shared between WHT and the tea powder-treated groups, while WHT, MTC, CPH, RBT, and JST contained 1, 14, 30, 14, and 10 unique metabolites, respectively (Fig. 3e). In terms of the total number of detected metabolites, MTC (1708) and JST (1706) had the highest numbers, whereas WHT had the lowest (1476).

Multivariate statistical analysis further confirmed the metabolic differences caused by tea powder addition. In the PCA score plot, WHT was clearly separated from MTC, CPH, RBT, and JST, and PC1 and PC2 together explained 48.75% of the total variance (Fig. 3f). OPLS-DA showed a separation trend similar to that observed in PCA (Fig. 3g), and the permutation test indicated that the model was stable and not overfitted (R^2^ = 0.002, Q^2^ = −0.3; Fig. 3h). Based on the OPLS-DA model and using VIP > 1 as the screening criterion, a total of 185 differential metabolites were obtained (Fig. 3i). Compared with WHT, 72–94 metabolites were upregulated and 11–25 metabolites were downregulated in the tea powder-treated groups. KEGG enrichment analysis showed that the differential metabolites were mainly enriched in flavonoid biosynthesis and flavone and flavonol biosynthesis pathways (Fig. 3j–m). This result indicates that tea powder addition mainly reshaped the non-volatile metabolic characteristics related to phenylpropanoid/flavonoid metabolism, which is consistent with the increases in total flavonoids, total phenolics, and antioxidant capacity.

### 3.4 Screening and pathway analysis of differential non-volatile metabolites

**Fig. 4:**
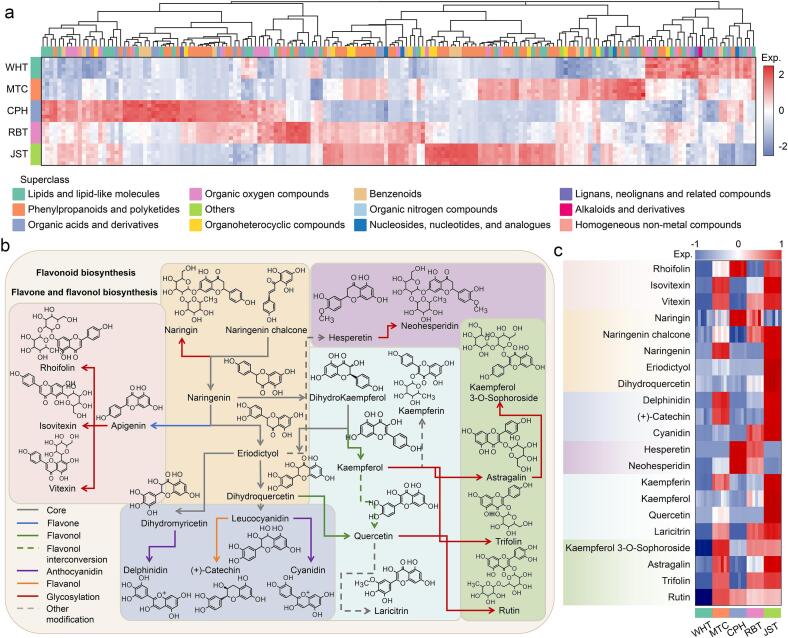
Classification characteristics of differential metabolites and pathway analysis in noodles with different tea powder treatments. (a) Heatmap and classification of differential metabolites. (b) Pathway annotation of differential metabolites involved in flavonoid biosynthesis and flavone and flavonol biosynthesis. (c) Hierarchical clustering heatmap of flavonoid-related differential metabolites.

The accumulation patterns of differential metabolites were further analyzed by heatmap. As shown in Fig. 4a, the metabolites were classified into 12 categories, among which Phenylpropanoids and polyketides contained the largest number of differential metabolites (59), followed by Lipids and lipid-like molecules (37). After the addition of different tea powders, the relative abundance of most differential metabolites showed an increasing trend, especially those belonging to Phenylpropanoids and polyketides, which generally exhibited higher accumulation in all tea powder-treated groups. This was mainly attributed to the abundant endogenous polyphenolic substances present in the tea powder raw materials. It is worth noting that the types and magnitudes of upregulated metabolites differed markedly among the tea powder-treated groups, which may be related to differences in the intrinsic metabolic composition of tea powder raw materials as well as their processing methods, thereby further affecting the metabolic specificity in noodles. To further characterize the distribution patterns of these differential metabolites at the pathway level (Fig. 4b), a total of 21 metabolites were annotated in Flavonoid biosynthesis and Flavone and flavonol biosynthesis (Table S4).

Specifically, in the upstream part of the flavonoid biosynthesis pathway, the chalcone intermediate naringenin chalcone serves as the precursor for the formation of flavanones, such as naringenin, eriodictyol, and hesperetin, as well as their glycosylated derivatives (naringin and neohesperidin). Heatmap clustering analysis (Fig. 4c) showed that different tea powder treatments significantly affected the accumulation pattern of metabolites in the flavanone branch. The JST treatment mainly retained relatively high levels of flavanone aglycones, particularly naringenin and eriodictyol. In contrast, the CPH treatment showed significant enrichment of flavanones and their glycosylated products, including naringin, neohesperidin, and hesperetin. In addition, the RBT treatment exhibited relatively high retention of naringenin chalcone. These results indicate that the addition of different tea powders led to distinct accumulation patterns of flavanone-related compounds along the biosynthetic pathway.

In the flavone branch, the abundances of representative flavones and their C-/O-glycosides, including vitexin, isovitexin, and rhoifolin, also showed significant differences among treatments. Among them, the JST treatment significantly increased the accumulation levels of vitexin and isovitexin, whereas CPH showed the most pronounced enrichment of rhoifolin. Previous studies have shown that flavone glycosides in tea widely occur in the form of C-glycosides and O-glycosides, and their changes during processing are closely related to flavor quality formation (Ke et al., 2025). The divergence between vitexin/isovitexin and rhoifolin among different treatment groups may reflect differences in the flavone glycoside composition of different tea powder raw materials.

In the downstream part of the pathway, dihydroflavonols represented by dihydroquercetin constitute a key hub connecting the flavonol, anthocyanin, and flavan-3-ol branches. Pathway topology analysis showed that this metabolism encompassed flavonols (quercetin, kaempferol, and laricitrin) and their glycosylated derivatives (rutin, astragalin, trifolin, and kaempferol 3-O-sophoroside), and further extended to anthocyanins (delphinidin and cyanidin) and flavan-3-ols, such as (+)-catechin. Overall, JST showed relatively high accumulation of quercetin, kaempferol, delphinidin, cyanidin, (+)-catechin, and some glycosylated flavonols; MTC specifically enriched flavonol glycosides such as rutin and kaempferol 3-O-sophoroside; whereas RBT exhibited relatively high accumulation of certain anthocyanin- and flavonol-related metabolites, such as cyanidin and laricitrin. Combined with previous reports on the dynamic changes of polyphenolic substances during white tea and black tea processing, these differences indicated that the variation among tea powder treatments in the downstream flavonoid branches was reflected not only in total levels, but also in the distribution patterns of flavonol glycosides, anthocyanins, and flavan-3-ol-related metabolites (Liu et al., 2022; Wang, Liu, et al., 2024; Wang, Xie, et al., 2024).

Further analysis from the perspective of raw material characteristics suggested that MTC, as the matcha-derived treatment, showed more pronounced enrichment of flavonol glycosides, which may be related to the relatively high flavonoid content in matcha and green tea, as well as the significant effects of cultivar and processing methods on flavonoid composition and astringency-related metabolic characteristics (Xue et al., 2025). CPH showed more pronounced enrichment of flavanone glycosides and some flavone glycosides, which was consistent with the compositional characteristics of citrus peels such as chenpi, which are rich in flavanone glycosides including naringin and neohesperidin (Matsuo et al., 2022; Shah et al., 2024). RBT showed relatively high accumulation of some anthocyanin- and flavonol-related metabolites, which may be associated with the relatively abundant cyanidin and its derivatives in rose raw materials, as well as the redistribution of flavonoid-related metabolites during black tea processing (Liu et al., 2022; Prakash et al., 2024). Overall, the compositional differences in flavonoid-related metabolic pathways among different tea powder treatments essentially resulted from the inherent chemical heterogeneity of tea powder raw materials, together with the selective retention and transformation of related components during noodle processing, such as matrix interactions and heat treatment. To further validate the reliability of the untargeted LC-MS results, eight representative phenolic/flavonoid-related non-volatile metabolites were quantified using reference standards (Table S5). The calibration curves of the eight reference compounds showed good linearity, with R^2^ values ranging from 0.9916 to 0.9973. Compared with WHT, tea powder incorporation increased the contents of most selected metabolites. Epigallocatechin increased from 149.58 ng/mg in WHT to 2817.97 ng/mg in MTC and 2985.13 ng/mg in JST. Rutin was most abundant in MTC, reaching 1085.15 ng/mg, which was approximately 11.49-fold higher than that in WHT. Hesperetin was mainly enriched in CPH, with a content of 272.28 ng/mg, approximately 47.91-fold higher than that in WHT. Naringin was also higher in tea powder-treated groups than in WHT, particularly in CPH and RBT. In addition, catechin, kaempferol, vitexin, and quercetin showed increased levels in one or more tea powder-treated groups. These targeted quantitative results confirmed the accumulation of representative tea-derived phenolic/flavonoid-related metabolites and supported the flavonoid-related pathway changes revealed by untargeted LC-MS analysis.

### 3.5 Volatile metabolic characteristics of noodles

**Fig. 5:**
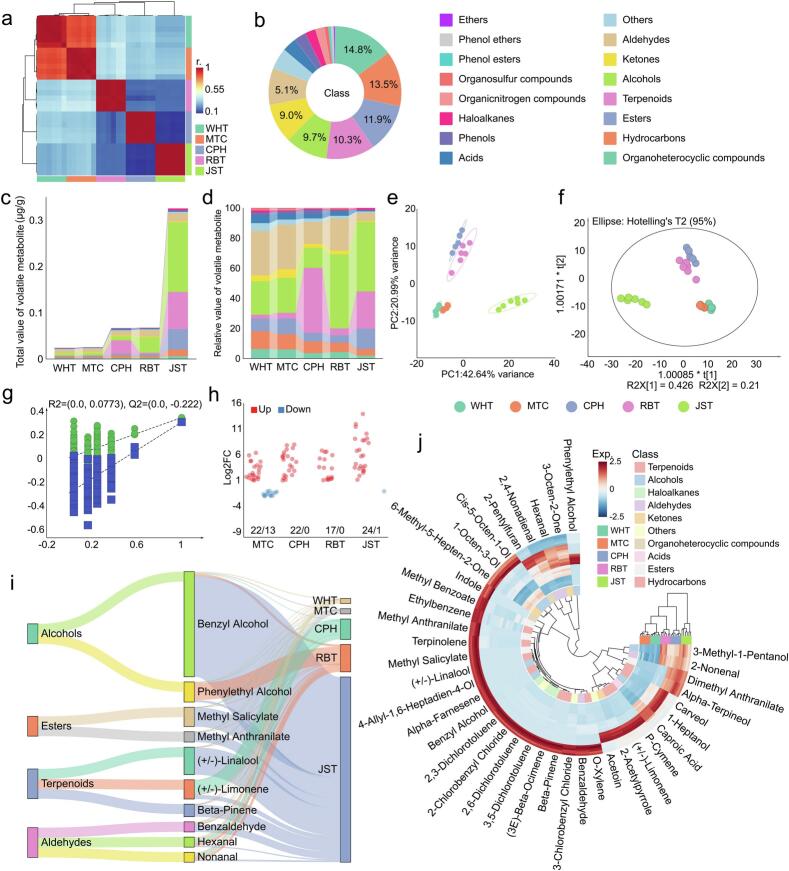
Effects of different tea powder additions on the volatile compound profiles of noodles. (a) Correlation heatmap. (b) Chemical category composition. (c) Total volatile compound content. (d) Relative composition of different classes of volatile compounds. (e) PCA score plot. (f) OPLS-DA score plot. (g) Permutation test. (h) Number of differential volatile compounds. (i) Composition analysis of the top 10 most abundant volatile compounds. (j) Hierarchical clustering heatmap of differential volatile compounds selected based on VIP values.

To clarify the effects of tea powder addition on the aroma characteristics of noodles, volatile compounds in WHT, MTC, CPH, RBT, and JST were analyzed by HS-SPME-GC–MS. Correlation analysis showed clear clustering among sample groups and high within-group correlations, indicating good data reproducibility (Table S6). Among them, WHT and MTC were clustered together first, suggesting that these two samples shared relatively similar volatile characteristics, whereas CPH, RBT, and JST differed markedly from them (Fig. 5a). In terms of chemical classes, the detected volatile metabolites were classified into 15 categories, among which organoheterocyclic compounds showed the highest proportion (14.8%), followed by hydrocarbons (13.5%), esters (11.9%), terpenoids (10.3%), alcohols (9.7%), ketones (9.0%), and aldehydes (5.1%). These seven categories together accounted for 74.3% of the total volatile components (Fig. 5b). Significant differences were observed in the total content of volatile metabolites among samples, with JST showing the highest total volatile content (0.33 μg/g), which was markedly higher than that of the other samples (Fig. 5c). CPH and RBT were at intermediate levels, whereas WHT and MTC showed the lowest contents (0.024–0.026 μg/g). The addition of different tea powders not only affected the accumulation level of volatile components in noodles, but also significantly altered their compositional characteristics. Specifically, WHT and MTC exhibited similar volatile profiles, both being dominated by aldehydes (29.4%–29.5%) and alcohols (22.2%–23.2%), together with certain proportions of hydrocarbons and esters (Fig. 5d). In contrast, terpenoids were markedly enriched in CPH, accounting for more than 43% and becoming its predominant volatile category. RBT was dominated by alcohols (49.2%), while maintaining a relatively high proportion of aldehydes (21.5%). JST not only exhibited the highest total volatile content but was also mainly composed of alcohols (46.2%) and terpenoids (24.7%), together with a relatively high proportion of esters (13.5%), which collectively distinguished its volatile metabolic characteristics from those of the other samples.

To further investigate the effects of different tea powders on the volatile metabolic profiles of noodles, PCA and OPLS-DA were performed for multivariate statistical analysis of the volatile compound data. The PCA results (Fig. 5e) showed that the first two principal components explained 42.64% and 20.99% of the total variance, respectively, with a cumulative explained variance of 63.63%, indicating that they adequately reflected the overall differences among samples. The samples within each group were closely clustered in the score plot, indicating good within-group reproducibility, while clear separation was observed among different treatments. In particular, WHT and MTC were positioned closest to each other in the score space, CPH and RBT were clearly separated from WHT, and JST was distinctly separated from the other samples along PC1, suggesting that its volatile metabolic profile was most strongly affected by tea powder addition. This trend was further strengthened in the supervised analysis, where the OPLS-DA score plot showed clearer sample separation (Fig. 5f), and all samples were distributed within the 95% Hotelling's T^2^ confidence ellipse. To evaluate the robustness of the OPLS-DA model, a 200-times permutation test was conducted. The results showed that the intercepts of R^2^ and Q^2^ were 0.0773 and − 0.222, respectively (Fig. 5g), indicating that the model did not show obvious overfitting. Based on the OPLS-DA model, differential volatile compounds were screened, and the numbers of differential compounds in each treatment group are shown in Fig. 5h. The results showed that, compared with WHT, all tea powder treatments induced changes in volatile metabolites. Specifically, 35, 22, 17, and 25 differential volatile compounds were identified in MTC, CPH, RBT, and JST, respectively, relative to WHT. Overall, most of the differential compounds showed increased abundance, indicating that tea powder addition generally promoted the accumulation of volatile components. Notably, JST not only exhibited a relatively large number of differential volatile compounds, but also showed greater Log_2_FC values, suggesting that it exerted the most pronounced effect on the volatile metabolism of noodles.

Analysis of the top 10 most abundant volatile compounds (Fig. 5i) showed that the dominant components were mainly distributed among alcohols, terpenoids, esters, and aldehydes, including benzyl alcohol, phenylethyl alcohol, methyl salicylate, methyl anthranilate, (±)-linalool, (±)-limonene, beta-pinene, benzaldehyde, hexanal, and nonanal. These compounds were mainly composed of terpenoids as well as aromatic alcohols, esters, and aldehydes, together with some aliphatic aldehydes, indicating that the samples were generally characterized by floral, fruity, and fresh notes, accompanied by certain green and fatty notes. Among them, linalool, phenylethyl alcohol, and benzyl alcohol were mainly associated with floral and sweet aromas, limonene and β-pinene contributed to fresh fruity and woody notes, whereas hexanal and nonanal were generally related to green and fatty notes (An et al., 2025; Kang et al., 2019; Liang et al., 2023).

Furthermore, a hierarchical clustering heatmap was generated based on VIP-selected differential volatile compounds (Fig. 5j) (Table S7). The results showed marked differences in the accumulation of key differential volatile compounds among treatment groups, and the clustering trend of samples was generally consistent with the PCA and OPLS-DA results. These differential compounds involved terpenoids, alcohols, aldehydes, ketones, esters, acids, hydrocarbons, and organoheterocyclic compounds, among which benzyl alcohol, phenylethyl alcohol, (±)-linalool, methyl salicylate, methyl anthranilate, terpinolene, alpha-farnesene, alpha-terpineol, carveol, and 2-nonenal contributed greatly to the discrimination among groups. Overall, compared with WHT, the tea powder-treated groups were enriched in more characteristic volatile compounds, indicating that tea powder addition not only increased the abundance of some volatile components, but also altered the composition of the major aroma compounds. Based on the odor attributes of these differential compounds, it can be inferred that the addition of tea powder may shift the aroma of noodles from a relatively simple cereal-like note toward a more complex composite aroma combining floral, fruity, fresh, and tea-like notes, in which (±)-linalool, methyl salicylate, methyl anthranilate, benzyl alcohol, and phenylethyl alcohol may be important contributors (Chen et al., 2024; Yan et al., 2024; Zhang, Lei, et al., 2025).

### 3.6 Effects of tea powder addition on characteristic volatile metabolites in noodles

**Fig. 6:**
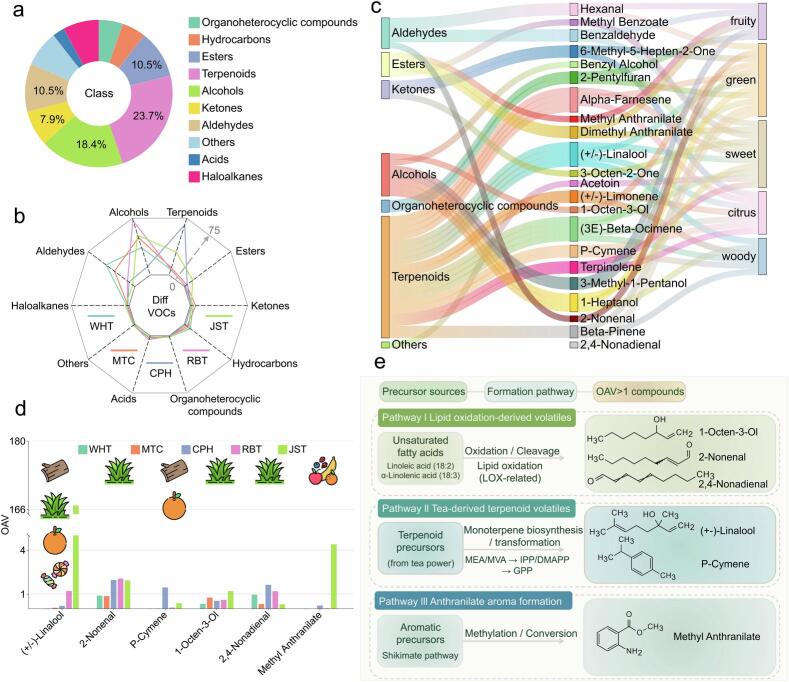
Effects of tea powder addition on characteristic volatile metabolites in noodles.

Differential volatile metabolite analysis showed that the volatile compounds in noodles were classified into 10 categories, among which terpenoids (23.7%), alcohols (18.4%), and aldehydes (10.5%) were the major components (Fig. 6a). The distribution of these categories differed markedly among treatments. WHT was mainly composed of aldehydes (38.93%) and alcohols (34.63%), while MTC was also dominated by alcohols (48.34%) and aldehydes (29.25%), indicating that the differential volatiles in these two groups were still primarily based on the aldehydes and alcohols commonly found in cereal-based products (Feng et al., 2024). In contrast, CPH was dominated by terpenoids (65.44%), RBT showed a high proportion of alcohols (72.04%), and JST contained relatively high proportions of alcohols (50.28%), terpenoids (26.03%), and esters (13.64%), suggesting a more diverse class composition. Meanwhile, aldehydes generally decreased in the tea powder-treated groups, accounting for only 4.02% in JST (Fig. 6b). These results indicate that tea powder addition not only altered the abundance of differential volatiles, but also affected their class distribution.

The Sankey diagram further illustrated the relationships between differential volatiles and their odor attributes (Fig. 6c). Among them, terpenoids comprised the largest number of differential compounds, including alpha-farnesene, (+/−)-linalool, (+/−)-limonene, (3E)-beta-ocimene, p-cymene, terpinolene, and beta-pinene. These compounds are commonly associated with citrus, floral, sweet, green, and woody odor notes in tea, and are therefore more likely to serve as important sources of freshness and tea-like aroma after tea powder addition (Liao et al., 2024; Wang, Gao, et al., 2024). In comparison, alcohols and aldehydes were more closely related to green, fruity, and fatty odor backgrounds, whereas esters were more likely to contribute fruity and sweet notes. Therefore, the increased proportion of esters in JST was more likely associated with enhanced fruity-sweet perception. Overall, the odor attributes corresponding to the differential volatiles were mainly green and sweet, followed by citrus, fruity, and woody (Xu et al., 2025; Zhang et al., 2025a). This also suggests that tea powder addition was more likely to promote the formation of a more layered and complex aroma in noodles.

OAV analysis further demonstrated that the aroma differences among treatments were mainly determined by a few odor-active compounds. Six key volatiles with OAV > 1 were identified, namely (+/−)-linalool, 2-nonenal, p-cymene, 1-octen-3-ol, 2,4-nonadienal, and methyl anthranilate (Fig. 6d). In WHT and MTC, all OAV values were below 1, indicating that their aroma was still mainly influenced by the original volatile profile. Nevertheless, the aroma contribution was mainly derived from 2-nonenal and 2,4-nonadienal, which are associated with green/fatty notes. In CPH, p-cymene (1.44), 2-nonenal (1.95), and 2,4-nonadienal (1.62) exceeded the threshold, suggesting that this treatment retained lipid oxidation-related notes while also exhibiting citrus-like characteristics. In RBT, (+/−)-linalool (1.21), 2-nonenal (2.05), and 2,4-nonadienal (1.19) indicated that this treatment combined floral and green features. By contrast, JST showed the most pronounced change, with the OAV of (+/−)-linalool reaching 166.78 and methyl anthranilate reaching 4.39, indicating that floral and sweet notes had become the major odor characteristics in JST (Gu et al., 2025).

The schematic diagram of the origins of the six key volatiles further helped explain the chemical basis of these changes (Fig. 6e). Among them, 2-nonenal, 2,4-nonadienal, and 1-octen-3-ol are mainly lipid oxidation-related volatiles (Huang et al., 2025). (+/−)-Linalool and p-cymene are more closely associated with terpenoid volatiles in tea powder and correspond to floral, fresh, and citrus-like characteristics. Methyl anthranilate represents the contribution of sweet- and fruity-type aromatic esters (Jin et al., 2024). These findings indicate that the effect of tea powder addition on noodle aroma was not simply due to the introduction of new volatiles, but rather to changes in the sensory contribution of different volatile compounds on the basis of the original lipid oxidation-related notes. The marked increases in linalool and methyl anthranilate in JST suggest that this treatment was more favorable for forming an aroma profile characterized by floral, sweet, and tea-like notes. The OAV of linalool exceeding 1 in RBT indicates a clearer floral character in this treatment. Although CPH did not show an obvious floral-dominant characteristic, the OAV of p-cymene exceeding 1 suggests that it more readily imparted fresh, citrus-like, and slightly herbal/woody aroma features to noodles. Overall, tea powders from different sources altered the contribution of different volatile compounds to noodle aroma, causing the aroma to shift from a relatively simple cereal-like profile toward a composite aroma characterized by floral, fruity, and fresh notes. These changes in odor-active compounds helped explain the increased aroma scores and overall acceptability of tea powder-treated noodles.

(a) Category distribution. (b) Radar chart showing category proportions. (c) Sankey diagram illustrating the relationships among categories, representative compounds, and their odor attributes. (d) OAV values for six key volatile compounds. (e) Schematic diagram of the potential sources of the six key volatile compounds.

## 4 Conclusions

This study demonstrated that tea powder has considerable potential for the development of fresh noodles, and that different tea powders can, to varying degrees, improve their quality characteristics and enhance the functional properties of fresh noodles. The incorporation of tea powder promoted the formation of a denser starch–gluten matrix, improved the cooking performance, textural properties, and sensory acceptability of fresh noodles, and increased the contents of starch and phenolic compounds as well as *in vitro* antioxidant activity. Metabolomic analysis further showed that tea powder treatment significantly affected both the non-volatile and volatile metabolic profiles of fresh noodles. The differences in non-volatile metabolites were mainly associated with changes in flavonoid-related metabolic pathways, while the volatile flavor profile shifted from a simple cereal-like aroma to more diverse aromatic characteristics. Future studies should further verify antioxidant-related mechanisms, evaluate the bioaccessibility and digestive stability of tea-derived active compounds using digestion models, and determine the optimal addition level of each tea powder through dose–response experiments. This study provides a reference for the selection of tea powder raw materials and the development of tea powder-enriched fresh noodles.

## CRediT authorship contribution statement

**Haozhen Li:** Writing – original draft, Methodology, Investigation, Funding acquisition, Data curation. **Xiangjun Chen:** Writing – review & editing. **Shuyao Wang:** Writing – review & editing. **HuiHui Zhao:** Writing – review & editing. **Yi Xie:** Writing – review & editing. **Long Yang:** Writing – review & editing, Writing – original draft, Supervision, Project administration, Investigation, Funding acquisition, Data curation, Conceptualization.

## Ethical statements

This institution has not established a Human Ethics Review Committee. Under current Chinese regulations, formal ethical approval is not required for sensory evaluation of such studies. All noodle samples were confirmed to be safe for consumption. Prior to testing, all assessors signed written informed consent forms after being informed of the study procedures and their rights, and participated voluntarily with the right to withdraw at any time.

## Funding

This work was supported by the Foundation of Innovation Team Project for Modern Agricultural Industrious Technology System of Shandong Province (SDAIT-25-01) and the National Natural Science Foundation of China (52500134). And we thank Supercomputing Center in Shandong Agricultural University for technical support.

## Declaration of competing interest

The authors declare no known competing financial interests or personal relationships that could have appeared to influence the work reported in this paper.

## Data Availability

Data will be made available on request.
